# A Hybrid System for Distinguishing between Brain Death and Coma Using Diverse EEG Features

**DOI:** 10.3390/s19061342

**Published:** 2019-03-18

**Authors:** Li Zhu, Gaochao Cui, Jianting Cao, Andrzej Cichocki, Jianhai Zhang, Changle Zhou

**Affiliations:** 1 Cognitive Science Department, Xiamen University, Xiamen 361005, China; 31520140154367@stu.xmu.edu.cn; 2 National Institute of Advanced Industrial Science and Technology, Tsukuba, Ibaraki 305-8560, Japan; gaochao.cui@aist.go.jp; 3 Department of Information System, Saitama Institute of Technology, Fukaya, Saitama 369-0203, Japan; cao@sit.ac.jp; 4 RIKEN Center for Advanced Intelligence Project, RIKEN, Nihonbashi, Tokyo 103-0027, Japan; 5 Skolkovo Institute of Science and Technology (Skoltech), 143026 Moscow, Russia; A.Cichocki@skoltech.ru; 6 Department of Informatics, Nicolaus Copernicus University, 87-100 Torun, Poland; 7 School of Computer Science and Technology, Hangzhou Dianzi University, Hangzhou 310018, China

**Keywords:** EEG, brain death, power spectrum density, entropy, CCA

## Abstract

Electroencephalography (EEG) signals may provide abundant information reflecting the developmental changes in brain status. It usually takes a long time to finally judge whether a brain is dead, so an effective pre-test of brain states method is needed. In this paper, we present a hybrid processing pipeline to differentiate brain death and coma patients based on canonical correlation analysis (CCA) of power spectral density, complexity features, and feature fusion for group analysis. In addition, time-varying power spectrum and complexity were observed based on the analysis of individual patients, which can be used to monitor the change of brain status over time. Results showed three major differences between brain death and coma groups of EEG signal: slowing, increased complexity, and the improvement on classification accuracy with feature fusion. To the best of our knowledge, this is the first scheme for joint general analysis and time-varying state monitoring. Delta-band relative power spectrum density and permutation entropy could effectively be regarded as potential features of discrimination analysis on brain death and coma patients.

## 1. Introduction

**Figure 1:**
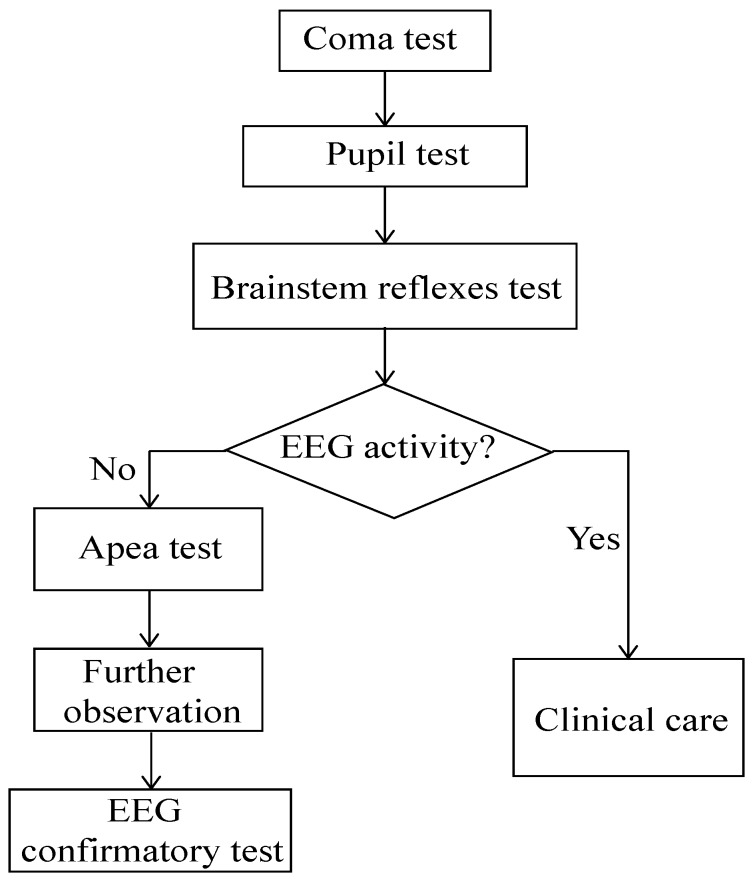
Procedure of brain death diagnosis.

The generally accepted definition of brain death is the irreversible loss functions of the whole brain (including hemisphere and brain stem) [1,2]. The procedure of brain death determination normally takes a long time and has certain dangerous operations (e.g., in apnea test, the respirator needs to be removed) [3] (see Figure 1). Electroencephalography (EEG) is frequently used to analyze and auxiliarily diagnose brain death clinically with the features of high time resolution and relative potable [4,5]. Since the characteristics of irreversible coma were first defined by the ad hoc Committee of Harvard Medical School, most countries have made their own legal provisions on brain death [6,7]. But the current criterion from EEG focuses on the amplitude value but is lacking in machine learning, signal processing, and statistical analysis investigations. In this paper, we aimed to use less electrodes to explore more distinctive results from EEG for brain death determination, specifically differentiating brain death from coma.

Power spectrum density (PSD) estimation is a signal processing measure to calculate a signal’s averaging power distributed across frequency. At present, quantitative analysis studies have indicated that neurology disorders cause power spectrum density to change in EEG signals [8,9,10]. For instance, Sury et al. have found that infants more than three months old had a higher power of EEG signal existing in the interval 5–20 Hz [11]. In Alzheimer’s patients the alpha EEG power is significantly decreased but the theta power is increased [12]. Myers et al. have discovered that the high-frequency brain activity in the beta-gamma range is preserved in the Cerebral Hypothermia population [13]. In addition, it consists of non-parameter and parameter-based methods [14]. Thereinto, the parametric autoregressive (AR) Yuler method is applied in our EEG PSD with the advantages of decreased spectral losses and better frequency resolution [15,16] compared to the classical Fast Fourier Transform (FFT). Another measure of signal processing is entropy, which characterizes the nonlinear dynamics in signals. The value of entropy represents the predictability of the signal and, specifically, the higher value means lower predictability [17,18]. Entropy measures have been used to learn the EEG behaviour in different neural disorder states, like anesthesia [19], schizophrenia [20], obsessive compulsive [21], epilepsy diagnosis [22], and Alzheimer’s disease [23]. Additionally, from different views of calculation, various entropy measures have been generated [17]. Thereinto, permutation entropy (PE) describes complexity through a phase space reconstruction with strengths of simplicity, robustness, constantness, and extremely computational efficiency [24,25]. Liang et al. have found that PE outperforms the other nine entropy measures in anesthesia EEG states detection [26]. To our best knowledge, PE has not been applied to brain death determination.

We hypothesize that a joint feature using both PSD and PE measures may improve the classification accuracy in brain death and coma patients. They represent the power energy distributions in patients in frequency domain and the complexity of EEG signals in time domain, respectively. The feature-based fusion method would combine the two features to improve the classification compared to the common concatenation EEG features. Such a hybrid system would be useful to design a computer-aided diagnosis system to address solutions germane to differentiating coma and brain death EEG. In this paper, our contributions are summarized as follows:

1. We developed a hybrid system for distinguishing between brain death and coma EEG of six symmetrical frontal electrodes that would be easier to implement with a computer-aided system.
2. We revealed the complementary characteristics of relative PSD and PE. PE is first introduced in studying such populations. And we obtained better performance by using CCA feature fusion.
3. We performed the AR-based time-frequency analysis and proposed the time-varying PE method to monitor the dynamical variations of the two features covering both frequency and time domains.

This paper is structured as follows: Section 1 describes the background of the paper. Section 2 reveals the methodologies in detail. Section 3 presents the experimental results. Finally, discussion and conclusion are given in Section 4 and Section 5.

## 2. Materials and Methods

### 2.1. Participants

The sample consisted of 53 patients’ EEG signals. The patients were from two groups, namely brain death and coma (in China, data recording of brain death patients is allowed). Of these, sixteen patients were diagnosed as brain dead (9 females and 7 male), with ages rangin from 17 to 85 years old. Thirty-seven patients were in coma status (9 females and 28 males), aged 23 to 65 years old. The diagnoses were made independently by two experienced clinicians, based on a rigorous diagnostic process. All patients were recruited from a Chinese hospital’s ICU from June 2004 to May 2012.

All subjects gave their informed consent for inclusion before they participated in the study. The study was conducted in accordance with the Declaration of Helsinki, and the protocol was approved by the Ethics Committee of Huashan hospital. In addition, given the coma state of the patient, the needed informed consent was obtained by the patient’s family, in order to perform the EEG recordings and to publish results based on their anonymized data.

### 2.2. EEG Recordings and Preprocessing

**Figure 2:**
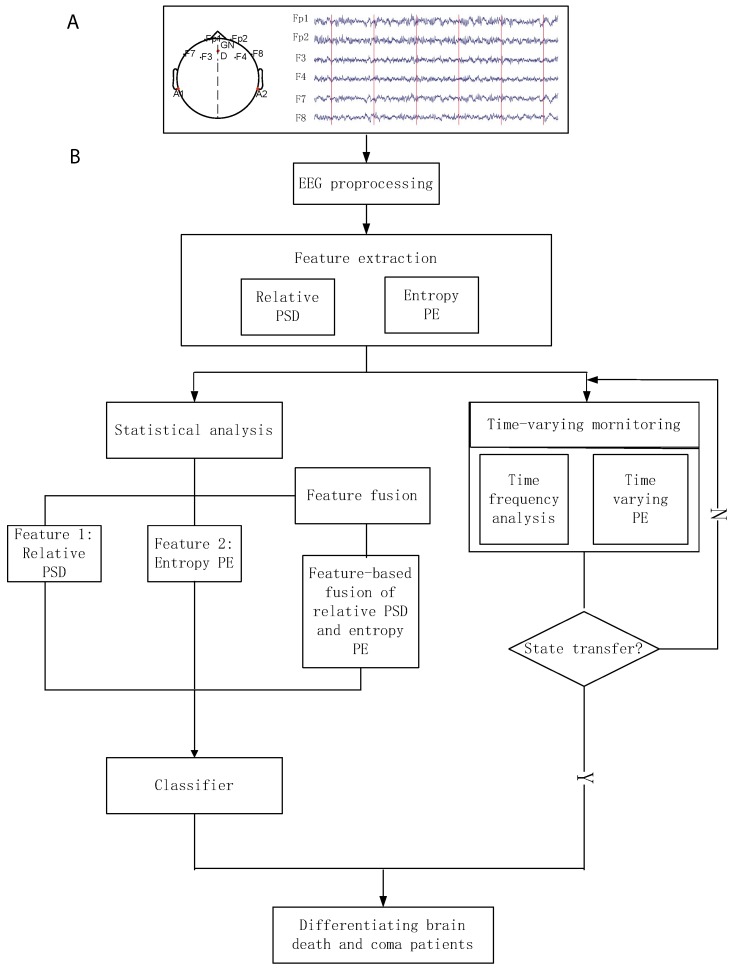
Processing pipe of classification between brain death and coma participants, using various features. (**A**) The lay of electroencephalography (EEG) electrodes and one patient’s EEG segmented with five non-overlapping windows. (**B**) The block diagram of discriminative analysis for these patients.

EEG data were recorded continuously, based on the patients’ health conditions. Particularly, the participants were recorded with different number sessions. EEGs were sampled at 1000 Hz using the NeuroScan ESI-64 system. During recording, the patients were laying on the ICU beds, and it was not safe to move them. So, we used forehead electrodes to avoid more hairy areas and touching of patients. The layout of nine electrodes is shown in Figure 2A. We segmented each EEG dataset into five 1-min non-overlapping epochs. Moreover, we used the ICA (independent component analysis)-based MARA plug-in in EEGLAB to remove the artifacts, and then filtered the denoised EEG into six sub-bands: γ2 (41–100 Hz), γ1 (30–40 Hz), β (13–30 Hz), α (8–12 Hz), θ (4–7 Hz), and δ (0.5–4 Hz). We explored the higher γ2 frequency as: (1) The bands of interest 0.5–100 Hz and (2) the potential effect of such bands in these populations.

### 2.3. Power Spectrum Density Estimation

#### 2.3.1. Relative PSD

The techniques can generally be divided into non-parametric and parametric methods. The parametric methods were proposed to increase the frequency resolution due to the windowed leakage effect suppression [14]. In EEG analysis, AR-based is the most common parametric method [27,28]. To obtain a better PSD estimation, we calculated it through a 250-points length sliding Hamming window with half overlapping. The function of AR(p) is as [8,29]:

(1) $$x\left(n\right)=\sum_{k=1}^{p}a_{k}x\left(n-k\right)+\sigma^{2},$$

where *p* is order of the model and $a_{k}$ is the coefficient.

We used the Yule-Walker method with minimization of a predictor error to estimate $a_{k}$. Then, the PSD is calculated as [30]:

(2) $$\hat{p}\left(f\right)=\frac{{\hat{\sigma }}_{\varepsilon }^{2}}{|1+\sum_{t=1}^{p}\hat{a}\left(i\right)e^{-jfi2\pi }|},$$

where $\hat{a}\left(i\right)$ are estimations of AR parameters obtained from the Levinson–Durbin recursions and p=10. The relative PSD is obtained as:

(3) $$P_{relative}=\frac{\sum_{f=f_{1}}^{f=f_{2}}P\left(f\right)}{\sum_{f=f_{l}}^{f=f_{h}}P\left(f\right)},$$

where $\left[f_{l},f_{h}\right]=\left[0.5,100\right]$ and $\left[f_{1},f_{2}\right]$ is the interval of specific sub-frequency band.

#### 2.3.2. Time-Varying Power Spectrum Estimation

Based on the power spectrum estimation by AR model, we applied the multi-taper approach to grasp the time-varying power spectrum estimation, which has been proven to be able to decrease variance further. The K orthogonal tapers divided the data into K AR models to obtain the spectrum estimation $\hat{P}\left(f\right)$, so the average spectrum $\bar{p(f)}$ is given by [31]:

(4) $$\bar{p(f)}=\frac{1}{K}\sum_{k=1}^{K}{\hat{P}}_{k}\left(f\right).$$

### 2.4. Entropy

#### 2.4.1. Permutation Entropy

PE was first proposed in 2002, which has been applied to data analysis in various fields. PE produces a time series into ordinal patterns that are coded by permutations [32]. PE has advantages of low computational complexity, stability, and simplicity [26]. For a given time series with *N* points x(1),x(2),...,x(N), the time series is reconstructed firstly:

(5) $$X_{i}=x(i),x(i+\tau ),...,x(i+(n-1)\tau ),i=1,2,...,N-\left(n-1\right)\tau .$$

So, we can get a group of symbol sequence from each row of the reconstructed matrix:

(6) $$s\left(l\right)=\left(j_{1},j_{2},...,j_{m}\right),$$

where l=1,2,...,k and k≤m!. There are m! permutations, and the reconstructed $X_{i}$ corresponds to one of those permutations. Suppose $P_{j}\left(j\in \left[1,k\right]\right)$ is the probability of *j*th symbol sequence. PE entropy is defined as:

(7) $$S_{1}^{\left(s\right)}=-\sum_{j=1}^{m!}P_{j}log\left(P_{j}\right).$$

#### 2.4.2. Time-Varying Permutation Entropy

To monitor the EEG’s complexity variation over time, we transferred the PE into the time-varying form. The values of PE are calculated in a set of consecutive time windows. We used *N*-1 windows to divide the EEG data $X_{N}$ into N intervals $x_{i}\left(n\right)$, and then calculate the PE in each *x*. For a given EEG, the definition of time-varying PE we made as:

(8) $$PE{\left(X_{N},m\right)}_{t}=\left[PE\left(x_{1}\left(n\right),m\right),PE\left(x_{2},m\right),...,PE\left(x_{N}\left(n\right),m\right)\right]=-\sum_{j=1}^{m!}P_{jt}log\left(P_{jt}\right),$$

where $P_{j}\left(j\in \left[1,k\right]\right)t$ is the probability of *j*th symbol sequence at t interval and *m* is the order of PE. As a result, if the denoted variable t ranges from $t_{1}$ to $t_{2}$ with a step length $t^{\prime }$, the values of PE are obtained in dynamic non-overlapping windows. We can use this PE entropy to monitor the time-varying PE values, with the state variation for clinical data.

### 2.5. Feature Selection

#### 2.5.1. Principle Component Analysis

Principle component analysis (PCA) is a feature selection method that projects the original feature matrix onto new space with lower dimension to avoid as much as the variation present in the original data [33]. These projections are called the principal components (PC) of the original dataset. In our study, only the first ($N_{a}$) and ($N_{b}$) PCs that explain at least 70% of the variance were retained for the relative PSD metrics and the PE metrics, respectively.

#### 2.5.2. Canonical Correlation Analysis

The reduced datasets given by the PCA output for the relative PSD matrices (A∈R) and PE matrices (B∈R) were combined to obtain a single set of features, which was realized by CCA-based feature fusion. We computed the canonical correlations of matrices A and B using the linear algebraic formulation presented in [34]. First, we performed a compact singular value decomposition (SVD) of matrices $A=U_{A}\sum_{A}V_{A}^{T}$ and $B=U_{B}\sum_{B}V_{B}^{T}$. Then the canonical projective matrices were computed as $W_{A}=V_{A}\sum_{A}^{-1}U$ and $W_{B}=V_{B}\sum_{B}^{-1}U$, where U and V were found as $U_{A}^{T}U_{B}=U\sum V^{T}$. As defined in Reference [34], feature fusion is achieved in the projected spaces by concatenation $X=AW_{A}$ and $Y=BW_{B}$:

(9) $$Z=\left(\begin{array}{cc} X & Y \end{array}\right)=\left(\begin{array}{cc} W_{A} & 0\\ 0 & W_{B} \end{array}\right),$$

where $Z\in R^{N_{p}\times 2r}$ with r=min(rank(A),rank(B)), are the canonical correlation discriminative features (CCDF).

### 2.6. Statistical Analysis

One-way ANOVA and post-correction were used to assess differences between groups of the relative PSD and PE for brain death and coma groups. Two observed returning values of ANOVA are *f*-value and *p*-value. Bigger *f*-value and lower *p*-value mean more significant difference [8,35]. Additionally, to minimize type I error, the strict statistical Bonferroni post-correction [36] was performed.

The final task was to apply the classifier to evaluate the performance of relative PSD and PE features on distinguishing between brain death and coma patients in a specific sub-band with significantly difference. The classifier adopted here was the support vector machine (SVM) implemented based on the LIBSVM library [37] and evaluated its performance by leave-one-out cross validation. The SVM was trained using a Gaussian Kernel. The kernel width and regulation parameter C in the SVM were tuned for each fold separately using a nested cross-validation 3-stage grid search. We quantify the performance of classifier based on the cross-validation stage results as:

$$\begin{array}{c} Sensitivity=\frac{TP}{TP+FN}\times 100\%\\ Specificity=\frac{TN}{FP+TN}\times 100\%\\ Acc=\frac{TP+TN}{TP+FN+FP+TN}\times 100\%, \end{array}$$

where *TP* is the number of brain death patients right classification; *TN* is the number of coma patients right classification; *FP* is the number of coma recognized into brain death; and *FN* is the number of brain death recognized as coma. Further, we also investigated the area under receiver operating characteristic (ROC) curve (AUC), which also indicates the performance of a classifier [38,39]. Additionally, Figure 2 shows the processing pipe for differentiating brain death and coma patients in EEG.

## 3. Results

### 3.1. Power Spectrum Density

#### 3.1.1. Sub-Bands PSD Ratio

**Figure 3:**
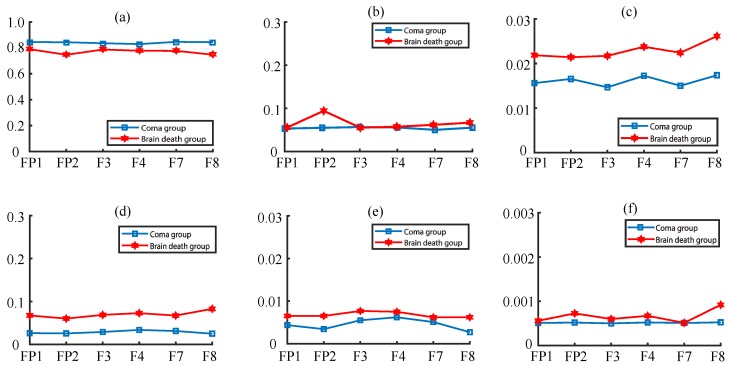
The total relative power spectrum density (rPSD) values of brain death and coma in (**a**) δ, (**b**) θ, (**c**) α, (**d**) β, (**e**) γ1, and (**f**) γ2 bands with six EEG electrodes.

We investigated the relative PSD (rPSD) features in group analysis and the time-varying spectral feature in special individual analysis over different frequency bands. Figure 3 shows the group feature analysis results. As the frequency rises, the rPSD is getting lower and the rPSD of lower band is accounting for large ratio between the two groups. For instance, rPSD values in δ band were at the interval [0.750.83], and the values in γ band greatly decreased into the the interval [0.00050.009]. The specific results are summarized as follows: (1) Across different frequency bands, the rPSD of coma group is higher only in θ band. (2) Across electrodes (Figure 3a,b) both in δ and θ bands, the rPSD values and the changing trends are similar, except FP2, F8 in β, and FP2 in the θ band. (3) Across electrodes in α and β bands (Figure 3c,d), the brain death’s rPSD values are much bigger, compared to the coma group. (4) Across electrodes (Figure 3e,f) in γ band, the rPSD values are similar in both groups with the increase of the frequency band.

**Figure 4:**
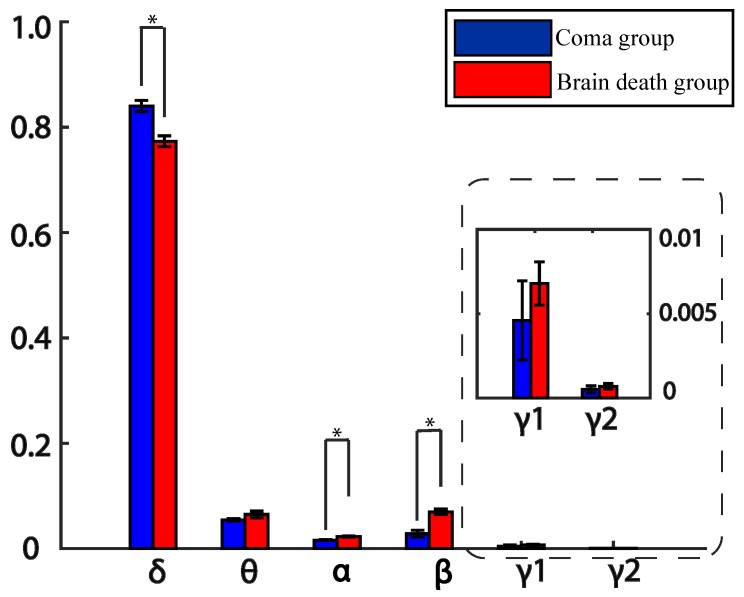
Mean rPSD values over six electrodes for sub-bands for brain death and coma patients. Error bars stand for standard deviations. Asterisk represents the significant difference with $p_{value}<0.01$ after ANOVA and post-correction.

Further, we averaged all the electrodes across frequency bands to investigate a deep knowledge of total rPSD characteristics. The results indicate that the dominate band is still δ band for both groups and the coma’s rPSD is higer in δ band, while the brain death’s rPSD values are higher in other bands like θ, α, β, γ1, and γ2. The rPSD values are very small for both groups in γ1 and γ2 bands (see Figure 4). Moreover, the rPSD of these two groups are significantly differences in $\delta (f_{value}=21.52$, $p_{value}$ = 9.1532 × 10${}^{-5}$), $\alpha (f_{value}=15.86$, $p_{value}=0.0015)$, and $\beta (f_{value}=17.10$, $p_{value}=0.0013)$ frequency bands after post-hoc correction (see Table 1).

#### 3.1.2. Time-Varying Power Spectrum Estimation

**Figure 5:**
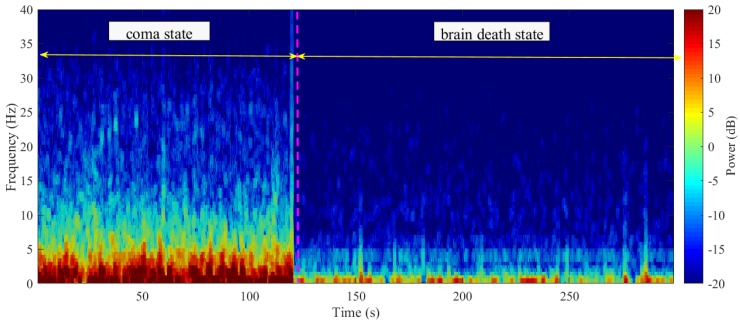
Comparison on time-varying power spectrum estimation for special case with coma and brain death state.

With regard to one special patient who experienced coma status turning into brain death state, four sessions of EEG data were recorded for this patient on 6 December 2011. Here, we took the discriminative channel FP2 as an example, according to the Figure 3, and calculated the multi-taper power spectrum estimation using the tapers in the range [23], 2 for window size and 0.1 for window step, consistent with the sampling frequency. In Figure 5, we show that, during coma state period, values of power spectrum are higher than brain death state period, especially in δ, θ and, α bands. The time-frequency analysis showed the state transferring through the time axis. We concluded that this previous coma participant’s state varied into quasi-brain death, in accordance with clinical diagnosis.

### 3.2. Entropy

#### 3.2.1. Permutation Entropy

**Figure 6:**
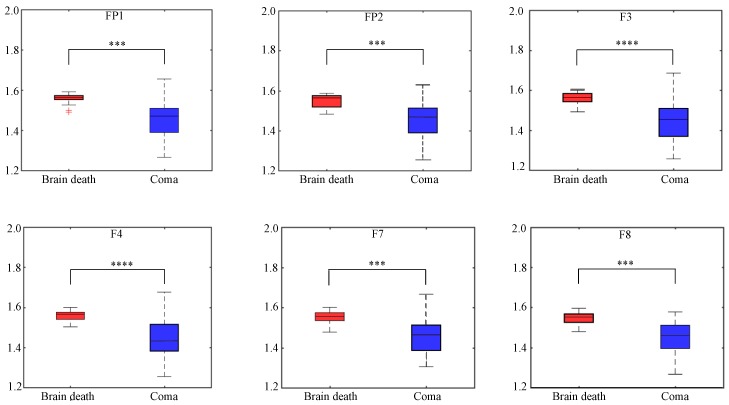
Comparisons of the permutation entropy (PE) across six electrodes for brain death and coma. Asterisks represent statistical levels returned by ANOVA and post-correction *** $p_{value}<0.001$, **** $p_{value}<0.0001$.

PE can be used to identify couplings between EEG series. The PE analysis results are indicated in Figure 6 and Table 2 across electrodes with embedding dimension of 20. The statistical post-correction is also used to show the significantly difference for the two groups with the marker asterisks for each channel. Obviously, compared to coma population, the mean values of brain death PE are bigger, while deviation values of brain death PE are smaller across electrodes. This indicates that: (1) The brain death EEG signals were unpredictably higher than the coma EEG signals (2) The PE values of the brain death group had a lower degree of dispersion than the coma group, even though there were two outliers in the FP1 electrode of the brain death group. Table 2 reveals the significantly different PE over all electrodes. The $p_{value}s$ were so superbly low (≤0.0005) that the PE across electrodes were discriminative features to classify brain death and coma patients’ EEG patterns.

**Figure 7:**
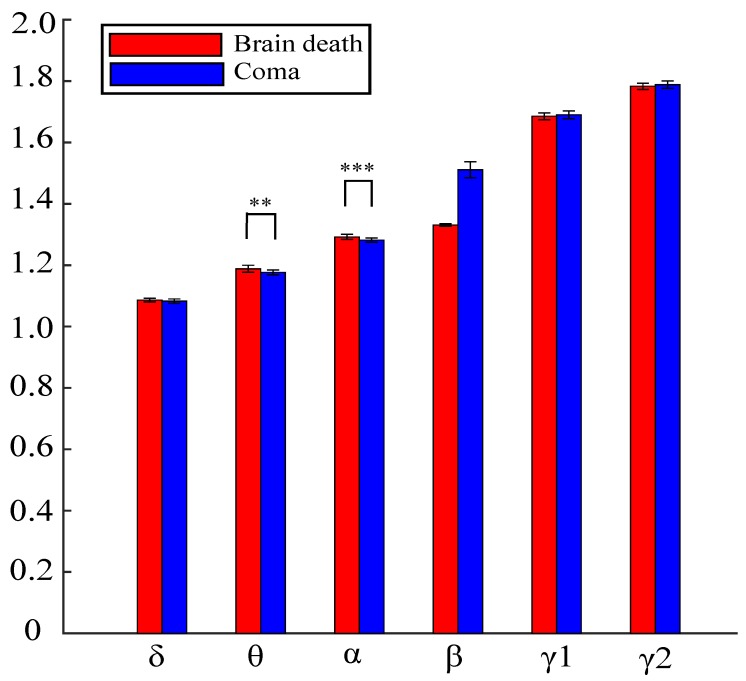
Comparisons on different sub-bands’ mean values of PE across six electrodes for the brain death and coma patients. Asterisks represent statistical significance levels returned by ANOVA and post-correction (** $p_{vlaue}<0.01$, *** $p_{value}<0.001$). Error bars represent standard deviations.

The statistical analysis results of PE for brain death and coma patients are further analyzed across different frequency bands, as shown in Figure 7 and Table 3. From Figure 7, it is clear that the PE values of brain death patients were found to be a little bit higher than that of coma group in δ, θ, and α bands. However, for the higher frequency bands, β, γ1, and γ2, the PE values of the brain death group are lower. And in Table 3, the statistical results indicate the PE significant differences between the two groups are in the θ and α frequency bands.

#### 3.2.2. Dynamic Permutation Entropy

**Figure 8:**
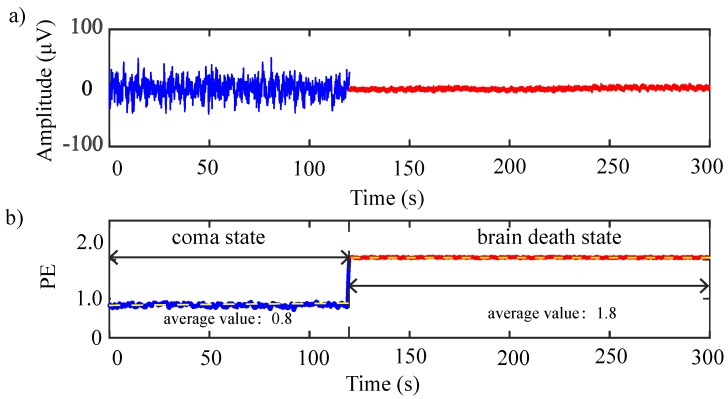
Time-varying PE averaged over the six electrodes for a special patient in two different states. (**a**) The averaged EEG signal over the six electrodes (**b**) The time-varying PE of the states variation. Dash lines indicate average value over duration.

For the same special patient analyzed in Section 3.1.2, we averaged the PE values across six electrodes to compare the patient in both brain death and coma states. From Figure 8b, the PE values of the patient in coma state were much lower than the patient’s values in brain death, where the variation is in accordance with the state transferring. We concluded that this previous coma participant’s state varied into quasi-brain death, in accordance with clinical diagnosis.

### 3.3. Feature Selection and Classification

We defined two different sets of features. First, a 6-dimensional feature set comprised of the overall rPSD in δ frequency band that yielded significant differences between groups in Table 1 and Figure 4. The second set was a 6-dimensional set comprised of the six electrodes PE values. For each of these feature sets, we performed PCA and retained the two first PC, as they explained at least 70% of the variance. Then, the CCA was performed using the resulting PC matrices for rPSD (A) and the PE (B) features.

To assess the performance of the proposed methodology, we used four reduced sets of features as follows:

set1: Two first PC for the rPSD,
set2: Two first PC for the PE values,
set3: Concatenation of the two first PC for both rPSD and PE values, and
set4: CCDF from Equation (9).

**Figure 9:**
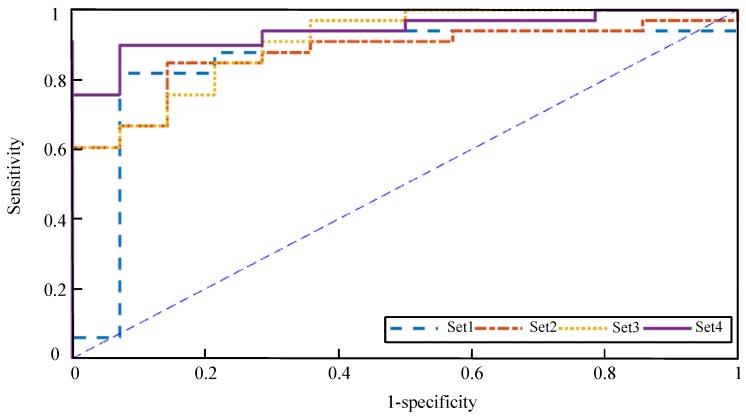
Comparison of the trade of between sensitivity and specificity. ROC curves are plotted to objectively compare the specificity and sensitivity of each classifier.

To optimize the parameters of SVM, we performed a 3-level grid search using exponentially growing sequences in the range [$2^{-5}$ $2^{15}$] for C and [$2^{-5}$ $2^{15}$] for gamma. Generalization of classification performances was ensured from leave-one-out cross validation. Classification performances of SVM classifier with each set of features are summarized in Table 4. The ROC curves for different classifiers are given in Figure 9. The area under each ROC curve is shown in the fifth column of Table 4. When SVM was trained using non-fused sets of features (set1 and set2), the classification rates were below 90% but above 85%, and the AUC values went above 85%. When we trained with the concatenation sets (set3 and and set4), the classification rates were improved. The best classification rate (92.3%) was achieved using the concatenation fusion of the features, yielding an AUC of 0.94. Compared with the accuracy rate using the concatenation set, the accuracy rate was improved using concatenation fusion (set4), which is related to the higher sensitivity.

## 4. Discussion

In this paper, we presented a methodological pipeline that aimed to differentiate the two groups patients. From the perspective of group analysis, we identified the most discriminative features for accurate classification with feature fusion technique using CCA over selected PC from EEG-based rPSD and PE values. From the view of individual analysis, we extended the power spectrum and PE to detect the power spectrum and complexity dissimilarity of patient’s EEG signals in different states. We chose PE to estimate the complexity because it has been proven to be an efficient entropy measure as it has less baseline variability and higher prediction probability [40].

Group analyses reveal that the dominate rPSD values of brain death (mean = 0.78, variance = 0.01) and coma (mean = 0.82, variance = 0.01) are in δ frequency band across electrodes, which suggests that brains in the two kinds of disorders show a slowing behavior. In addition, compared with the coma group, the rPSD values of brain death were decreased in δ band and significantly increased in α and β bands. The findings are in accordance with the PE values calculated in the time domain. The higher the PE is, the higher the complexity is. The PE values of brain death were higher than the coma group, so the distribution of different rhythm components was wider, whereas the dominate rPSD values were in δ band for the two groups, and the rPSD values of brain death were higher in α and β bands. Individual analysis showed that the time-frequency analysis and extended dynamic PE measure could be used to monitor the changing states within the same brain. For the same patient, the power spectrum values decreased and PE values increased when the patient moved into the brain death period.

It has also been found that the complexity measures of brain death are increased. Related works report similar results. For instance, Chen et al. discovered the significantly higher complexity values of brain death patients across electrodes by the approximate and normalized singular spectrum with time delay entropy measures [3]. Ni et al. have distinguished the quasi-brain death from brain death patients by the complexity values and the values of the brain death group were higher than quasi-brain-death patients using sample entropy [41]. Meanwhile, Shi et al. analyzed the power spectrum analysis and found the significant differences between coma and brain death groups. Further, the same group reported on case study of the spectral energy [42,43,44,45]. However, the previous works focus on one single feature analysis, like complexity and case study, without the classification analysis. Here, we attempted to differentiate brain death and coma groups using different classifiers and increasing the performance using concatenation rPSD with complexity PE, which is evaluated by ROC curves. The accuracies of concatenated and CCDF-based concatenated features are 90% (93.93% sensitivity; 81.81% specificity) and 93.5% (94.1% sensitivity; 84.62% specificity) respectively. While using the separate feature, the accuracies achieved 88.4% (91.55% sensitivity; 75% specificity) and 87.20% (90.63% sensitivity; 73.33% specificity) by rPSD and PE, respectively. Thus, the proposed concatenated CCDF-based feature would be a good discriminative feature to differentiate brain death patients from coma patients.

The limitations of our paper are illustrated as follows: (1) More classification verification needs more data. Currently, however, the statistical analysis would be effective to differentiate brain death and coma patients. (2) Most previous studies focused on comparing well-defined groups, such as healthy controls versus clinical population. Here, the data came from two clinical groups, which were only distinguishable based on whether they were brain dead.

## 5. Conclusions

This paper presented a system to differentiate brain death patients from coma patients. To our best knowledge, this is the first scheme for both general analysis and time-varying state monitoring based on relative PSD and PE processing in this population. In our experiments, we reached the highest accuracy using a method of feature fusion based on canonical correlation analysis.

The rPSD values of α and β bands increased, but δ and γ bands extremely decreased. Moreover, we investigated EEG signals’ complexity by PE across electrodes. It is shown that the complexity across electrodes in brain death group would be remarkably increased and PE value is significantly increased in θ and α bands. Finally, the best performance of classification was obtained by concatenated fusion feature of rPSD and PE. Therefore, we suggest that rPSD in δ band and PE would be discriminative EEG features for brain death and coma recognition.

If further validated, we would expect this approach to help in the estimation of brains in different level of consciousness and to provide a new research approach to characterize problems in populations with neurology disorders.

## Figures and Tables

**Table 1:** Statistical results of all sub-bands rPSD for these groups.

EEG Bands	$f_{\mathrm{value}}$	$p_{\mathrm{value}}$
δ	21.52	9.1532 × 10${}^{-5}$
θ	1.62	0.2094
α	15.86	0.0015
β	17.10	0.0013
γ1	2.04	0.1601
γ2	0.57	0.4543

**Table 2:** Statistical results of PE across electrodes between the brain death and coma groups.

EEG Electrode	$f_{\mathrm{value}}$	$p_{\mathrm{value}}$
FP1	17.53	9.1721 × 10${}^{-5}$
FP2	14.17	0.0005
F3	19.92	5.3619 × 10${}^{-5}$
F4	19.11	7.1912 × 10${}^{-5}$
F7	17.60	0.0001
F8	18.19	0.0001

**Table 3:** Statistical results of sub-bands’ PE across electrodes between the brain death and coma populations.

EEG Sub-Band	$f_{\mathrm{value}}$	$p_{\mathrm{value}}$
δ	0.08	0.772
θ	11.83	0.0013
α	16.57	0.0002
β	2.68	0.1089
γ1	0.87	0.3553
γ2	2.76	0.1036

**Table 4:** Accuracy, sensitivity, and specificity related with the proposed methodology.

Feature Set	ACC (%)	Sensitivity (%)	Specificity (%)	AUC
1	88.40	91.55	75.00	0.89
2	87.20	90.63	73.33	0.88
3	90.10	93.93	81.81	0.91
4	93.50	94.28	84.62	0.94
